# Changes in pediatric hospital care during the COVID-19 pandemic: a national qualitative study

**DOI:** 10.1186/s12913-021-06947-7

**Published:** 2021-09-11

**Authors:** Nicole Y. Penwill, Nadia Roessler De Angulo, Priya R. Pathak, Clairissa Ja, Martha J. Elster, Daniela Hochreiter, Jacqueline M. Newton, Karen M. Wilson, Sunitha V. Kaiser

**Affiliations:** 1 Department of Pediatrics, University of California, San Francisco, 550 16th Street, San Francisco, CA 94158 USA; 2 University of California, Davis, 1 Shields Ave, Davis, CA 95616 USA; 3 Lawrence and Memorial Hospital, 365 Montauk Ave, New London, CT 06320 USA; 4 Children’s National Hospital, 111 Michigan Ave NW, Washington, DC 20010 USA; 5 The Kravis Children’s Hospital at the Icahn School of Medicine at Mount Sinai, One Gustave L. Levy Place, Box 1198, New York, NY 10029 USA; 6 Philip R. Lee Institute for Health Policy Studies, 3333 California St, San Francisco, CA 94118 USA

**Keywords:** Child, hospitalized, COVID-19, Delivery of health care, Hospitals, community, Hospitals, pediatric, Qualitative research

## Abstract

**Background:**

The COVID-19 pandemic has necessitated rapid changes in healthcare delivery in the United States, including changes in the care of hospitalized children. The objectives of this study were to identify major changes in healthcare delivery for hospitalized children during the COVID-19 pandemic, identify lessons learned from these changes, and compare and contrast the experiences of children’s and community hospitals.

**Methods:**

We purposefully sampled participants from both community and children’s hospitals serving pediatric patients in the six U.S. states with the highest COVID-19 hospitalization rates at the onset of the pandemic. We recruited 2–3 participants from each hospital (mix of administrators, front-line physicians, nurses, and parents/caregivers) for semi-structured interviews. We analyzed interview data using constant comparative methods to identify major themes.

**Results:**

We interviewed 30 participants from 12 hospitals. Participants described how leaders rapidly developed new hospital policies (e.g., directing use of personal protective equipment) and how this was facilitated by reviewing internal and external data frequently and engaging all relevant stakeholders. Hospital leaders optimized communication through regular, transparent, multi-modal, and bi-directional communication. Clinicians increased use of videoconference and telehealth to facilitate physical distancing, but these technologies may have disadvantaged non-English speakers. Due to declining volumes of hospitalized children and surges of adult patients, clinicians newly provided care for hospitalized adults. This was facilitated by developing care teams supported by adult hospitalists, multidisciplinary support via videoconference, and educational resources. Participants described how the pandemic negatively impacted clinicians’ mental health, and they stressed the importance of mental health resources and wellness activities/spaces.

**Conclusions:**

We identified several major changes in inpatient pediatric care delivery during the COVID-19 pandemic, including the adoption of new hospital policies, video communication, staffing models, education strategies, and staff mental health supports. We outline important lessons learned, including strategies for successfully developing new policies, effectively communicating with staff, and supporting clinicians’ expanding scope of practice. Potentially important focus areas in pandemic recovery include assessing and supporting clinicians’ mental health and well-being, re-evaluating trainees’ skills/competencies, and adapting educational strategies as needed. These findings can help guide hospital leaders in supporting pandemic recovery and addressing future crises.

**Supplementary Information:**

The online version contains supplementary material available at 10.1186/s12913-021-06947-7.

## Introduction

The coronavirus disease 2019 (COVID-19) pandemic necessitated rapid changes in healthcare delivery in the United States, including the care of hospitalized children [1]. In the US, there are over 6 million pediatric hospitalizations annually, leading to nearly $50 billion in healthcare costs [2]. The COVID-19 pandemic caused rapid, dramatic changes in the epidemiology of pediatric hospitalizations as well as hospital operations (e.g., creating capacity for surges in patient volumes, instituting new infection control protocols) [3, 4]. Hospital leaders and front-line clinicians strived to maintain high-quality care for children despite the immense challenges of this novel pandemic.

Hospital leaders had limited resources to guide them in successfully navigating these challenges. The Hospital Disaster Resilience framework provided guidance on major domains of focus for promoting hospital resiliency: hospital safety, disaster preparedness and resources, continuity of essential medical services, recovery, and adaptation [5]. The Children’s Hospital Association published guidance on increasing adult capacity in general hospitals by consolidating pediatric care within specialized centers [6]. Hospital leaders also published editorials on general leadership principles, surge planning, and preparing clinicians to expand scope of practice [7–9]. However, these were not systematic studies, and they were limited to the experiences of large, academic centers and free-standing children’s hospitals, where < 30% of children are cared for nationally [10]. To our knowledge, there have been no multicenter studies of changes in pediatric healthcare delivery and lessons learned during the COVID-19 pandemic.

The objectives of this study were to identify major changes in healthcare delivery for hospitalized children during the COVID-19 pandemic, identify lessons learned from these changes, and compare and contrast the experiences of children’s and community hospitals. Our findings can help guide hospital leaders and clinicians in maintaining high-quality care for hospitalized children during this pandemic and in future crises.

## Methods

### Study design and population

For this qualitative study, we conducted semi-structured interviews of pediatric hospital administrators, front-line clinicians (physicians, nurses), and caregivers. We identified potential participants using purposeful sampling [11], focusing on the six states with the highest COVID-19 hospitalization rates during the first wave of the COVID-19 pandemic (March 1, 2020 - May 1, 2020): New York, New Jersey, Connecticut, Massachusetts, Virginia/District of Columbia, and Louisiana [12]. In each of these states, we identified a children’s hospital and a community/general hospital where we had direct or indirect contacts. We purposefully sampled a first participant who was knowledgeable about changes in inpatient pediatric care (all conditions). Our initial participant at each hospital then helped to identify additional participants (snowball sampling) [13]. We interviewed two participants from each hospital, seeking a mix of front-line clinicians and administrators/leaders in hospital operations. We also interviewed parents/caregivers from three hospitals. These participants were members of hospital family advisory councils and had children who utilized medical care during the pandemic. This study was reviewed and deemed exempt by the University of California, San Francisco Institutional Review Board.

### Theoretical framework

Two resources informed this study: the Hospital Disaster Resilience framework and the AcademyHealth Report: “Health Systems Respond to COVID-19: Priorities for Rapid-Cycle Evaluation.” [1, 5] The Hospital Disaster Resilience framework outlines major domains of hospital resiliency, as described above. The AcademyHealth Report identifies high-priority research questions for improving quality and safety of care during and after the COVID-19 pandemic. We used these resources to draft semi-structured interview guides (Additional file 1).

### Data collection and analysis

In preparation for conducting interviews, our senior author (SVK), who has conducted several prior peer-reviewed qualitative studies [14, 15], provided all members of our study team with interview training to ensure interview quality and consistency (e.g., prioritizing open-ended questions, utilizing probes). Other authors with qualitative interview experience (PRP, MJE, JN) contributed to this training session. Additionally, all interviewers watched a recorded video interview conducted by the senior author and reviewed transcripts of the first 3 interviews to finalize and refine interview strategies.

We conducted one-on-one interviews via videoconference. We used the first three interviews to analyze the interview process and modify the interview guide as needed. Verbal consent was obtained for participation, analysis, and dissemination of study findings. All interviews were recorded, transcribed, de-identified, and proofread for accuracy. We conducted interviews until saturation was achieved (sufficient data collected to fully explore important issues, and new data is repetitive) [16].

We analyzed interview data in Dedoose 8.3.45 (Manhattan Beach, CA) using constant comparative methods [16, 17]. We reviewed several interview transcripts to develop a preliminary codebook. Four authors (NYP, NRD, PRP, SVK) then simultaneously, but independently, coded 4 transcripts and compared codes to ensure agreement. We developed the code structure through an iterative, inductive process. Our whole team then examined the coded data together to finalize the codebook. We performed line-by-line coding, and every transcript was coded independently by at least two coders. Coders met periodically throughout the coding process to ensure consistency. Coding and analysis were conducted in parallel with interviews, so we could continue interviews until we reached saturation. We compared data from children’s and community hospitals to compare and contrast experiences. Data from the most frequently applied codes were systemically reviewed to develop memos and identify themes. Disagreements were resolved through review of primary data and iterative discussions among all study investigators. To ensure study rigor, we used reflexivity (study team members reflecting on how prior life experiences may impact the analytic process), member checking (soliciting study participant feedback), and triangulation (participants and investigators represented multiple roles, with unique perspectives: administrator, physician, nurse, caregiver, fellow, resident, student) [16].

## Results

We identified one children’s hospital and one community/general hospital in each state (total 12 hospitals). We interviewed a total of 30 participants (24 administrators/clinicians [2 participants from each hospital], and 6 parents/caregivers). We interviewed 9 front-line physicians and 15 participants with both clinical and administrative/leadership roles in hospital operations (9 physicians, 6 nurses).

Below, we summarize the major themes that emerged from our data, which fall into 3 broad categories: organizational changes, healthcare delivery for patients with COVID-19, and effects on hospital personnel. For each theme, we present an overview of the ideas and experiences of all study participants as well as 1–2 quotes from participants that exemplify that theme. Themes are presented in order of highest coded frequency. Table 1 provides illustrative quotes from study participants by theme.

**Table 1:** Illustrative Quotations for Each Theme

Theme	Exemplary Quote
Developing New Policies	“They setup a command center … my division leader, the Chair of Pediatrics, the Chair of Surgery, all the major division heads plus our quality improvement expert team, during the height of COVID in March/April, they were having daily meetings. We were getting daily updates from those meetings and policies were being made there.” –*Front-line physician, Hospital 1*
	“It really did add a whole other layer to COVID when you’re dealing with a mom and a baby. So, just trying to develop those policies on what’s going to keep the patients safe. What will keep the baby safe? What’s going to keep our staff safe?” –*Nurse administrator, Hospital 6*
Communicating with Hospital Staff	“I think for staff … the nurses and therapists, and [medical assistants], I think they could have done a better job with having … more consistent … opportunity for folks to ask questions and just be heard.” *–Physician administrator, Hospital 11*
	“Because in the very beginning [the situation] was evolving pretty quickly … in addition to the daily operational briefing, there were a lot of … meetings and phone calls, emails, just trying to make sure that everybody was aware and staying on the same page.” –*Front-line physician, Hospital 11*
Using Video Technology	“If we needed subspecialty consults and we didn’t feel like we needed to perform an actual physical exam, we would communicate that with our specialists, and they would [conduct] their consults via telehealth into the room, and then decide if they needed to actually go in or not.” –*Physician administrator, Hospital 1*
	“At our last inpatient stay, it was very clear we just weren’t going to see anybody … I do feel it was frustrating not to see anybody and be able to ask questions …. There were multiple things that I thought we would be able to talk to them about in his room and we would see them in person. That never happened.” –*Caregiver, Hospital 5*
Organizing Clinical Spaces and Supplies	“It probably was not a better idea to close all community sites down [to] centralize care because, in hindsight, it would have been better to keep the areas open that didn’t see as many cases and keep the care more decentralized and support the hospitals that were seeing higher surges.” *–Front-line physician, Hospital 2*
	**“**I think [being] part of a large system has really helped us. We have not had any issues with [personal protective equipment (PPE)] or reagent … we have never run out of tests. PPE, we’ve never had an issue with, and I think it’s being part of the larger system.” –*Nurse administrator, Hospital 2*
Newly Caring for Hospitalized Adults	“We were told that afternoon, we’re getting … somewhere between 7 to 12 [adult] patients. All of them complicated … I mean, we’d have support from the on-call medicine resident. Breaking that news was hard. Telling everybody it’s going to be okay was hard, because I was terrified. I was certainly terrified. It just felt like there was no plan in place.” –*Front-line physician, Hospital 9*
	“We’re caring for patients who are 30 and under, and then we have [a] list of exclusion criteria that we felt like those are medical issues that should not be cared for in a pediatric hospital, and that they should stay at the adult hospital.” –*Front-line physician, Hospital 11*
Testing Patients and Staff for COVID-19	“Given that we’re a community hospital and not part of a large university kind of setting...we now have good supply, but I think there’s just the concern that we will run out.” –*Physician administrator, Hospital 4*
	“One of the nurses let me know what their testing schedule was—we are tested on such and such a schedule so that’s how we know that we are safe to be here …. It was really helpful.” *–Caregiver, Hospital 5*
Navigating Staffing Challenges	“A number of nurses became sick with COVID. And it’s a small unit with a small staff and they didn’t have staff to support keeping the pediatric unit open.” –*Front-line physician, Hospital 6*
	“How do you do staffing when your volume is half of what it normally is? So many nurses got furloughed, so many people got furloughed during the height of this. Even when you’re on the frontline dealing with it, there’s furlough.” –*Front-line physician, Hospital 1*
Impacting Mental Health	“Dealing with death was really, really hard … One of the hospitalists that works with me in the community site [had] three [patients die] that week. That was the only week she was going to work. She couldn’t handle more.” –*Front-line physician, Hospital 2*
	“The amount of stress and anxiety that the staff had felt and the weariness, day after day, month after month, of the pandemic... I feel like the organization [had] really good support for employees to get counseling services …” –*Nurse administrator, Hospital 3*
	“I hated feeling that fear going [to the hospital] … We were thinking of tornadoes or hurricanes. I felt like I was flying into the eye of the storm that night when I shouldn’t be, I should be going the other direction.” *–Caregiver, Hospital 3*
Disrupting Trainee Education	“Many of [the medical students] didn’t even come to the hospital for three or four months. And now when they resumed their rotations, they are doing maybe five-week rotations or four-week rotations. It’s a very abbreviated sort of experience for them.” –*Physician administrator, Hospital 8*
	“We were talking about bronchiolitis as a hypothetical disease process, which is wild.” *–Frontline physician, Hospital 4*

### Organizational changes

We found fundamental hospital processes around policy development and communication were quickly and dramatically altered during the COVID-19 pandemic.

#### Developing new policies

**Fig. 1:**
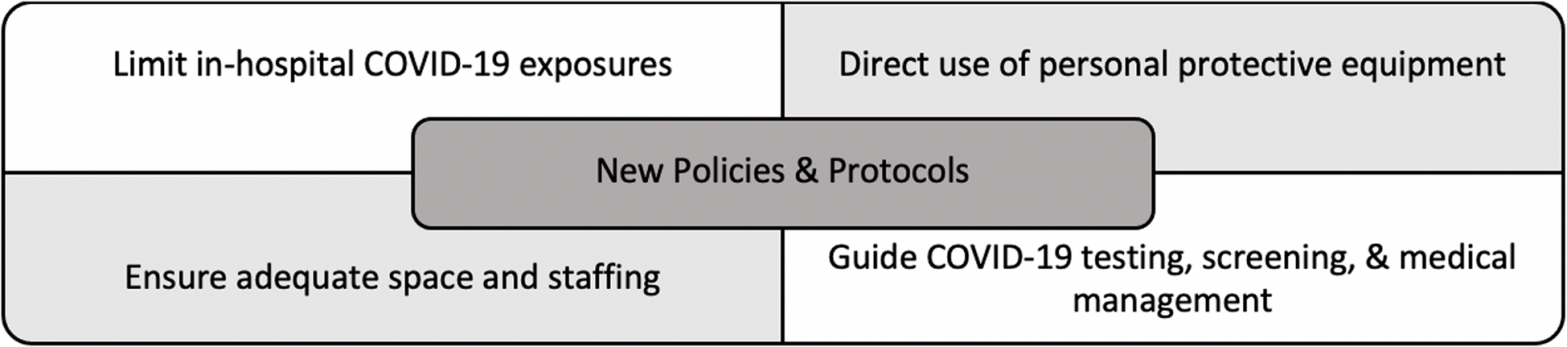
Four Domains of Developing Policies during COVID-19

Although many hospitals had pre-existing disaster plans/policies, these policies were typically designed for mass casualty events or natural disasters and had limited utility during the pandemic. Hospital leaders rapidly developed new policies in 4 main domains (Fig. 1).

Many institutions formalized integrated “command centers,” composed of core leadership and key multidisciplinary stakeholders. Command center personnel met daily (usually by videoconference) to review evolving data and outside agency recommendations, develop and update policies, make contingency plans, and streamline communication with hospital staff.

*“The coordination, communication, [and] overall top-level leadership was outstanding … protocols that were put in place for protection of staff and protection of patients felt really good to me as well. It felt like it was highly evidence-based, efficient.”* –Physician administrator, Hospital 5

Compared to participants from children’s hospitals, those from community hospitals reported feeling under-represented in the policy-making process. They also more often emphasized the challenges of developing policies and protocols related to maternal-newborn care.

*“I felt like being in a community hospital … exposed gaps in communication and flow of information to us as a smaller department within a bigger hospital … we were often not included in decision making.”* –Front-line physician, Hospital 2

Caregivers generally applauded robust COVID-19 infection control policies and reported that decreases in bedside visits by clinicians led to an enhanced “healing environment.” However, they reported hardships around decreased access to certain supports (e.g., child life services, kitchens) and visitation restrictions.

*“It's not an easy thing to do to hand over your child … to surgery, and then go sit in a waiting room by yourself. You have no one to lean on.”* –Caregiver, Hospital 3

Key Lessons: In future crises, hospitals may benefit from establishing a command center. In developing new policies, hospitals should utilize the most up-to-date evidence; create transparency in the policy-making process; and directly solicit input from stakeholders, including community hospital leaders.

#### Adapting communication strategies

Participants expressed that hospital leaders needed to establish frequent, effective communication with staff and utilized multiple modalities to do so. In addition to electronic mail, websites, and in-person visits to hospital units, videoconferencing increased dramatically to facilitate socially distanced meetings, town halls, and trainee education. Participants valued opportunities for real-time, bi-directional communication and feedback. However, participants sometimes felt overwhelmed by excessive e-mails and texts, particularly if the information was perceived as redundant, not actionable, or poorly organized.

*“One of the things that went really well was … daily town halls and transparency for the rapidly evolving situation.” –*Front-line physician, Hospital 3

Key lessons: For communicating with staff, hospital leaders should consider communicating frequently and regularly; utilizing multiple communication modalities (e.g., meetings, electronic mail); establishing bi-directional communication (ways for staff to communicate with leaders); and ensuring communication is simple, well-organized, and up to date.

In terms of communication with patients, hospitals widely utilized telemedicine to limit COVID-19 exposures and preserve personal protective equipment (PPE). Areas of new/increasing use included communication with patients/families, subspecialist consultation, and translation services. Clinicians and caregivers reported that telehealth reduced barriers to accessing care and improved care coordination, such as post-discharge follow-up. However, participants expressed concerns about decreases in communication quality (due to less direct interaction), particularly for non-English speakers and low-resourced families with limited access to needed technology.

*“[We were] forced to go to either phone translators or iPad translation services … it's a little bit less effective and obviously that differentially affects [non-English speakers].”*

*–*Physician administrator, Hospital 5

Key Lessons: Hospitals can leverage videoconference to limit infectious exposures and maintain personal protective equipment. However, these technologies may have limitations for non-English speaking patients, requiring optimization of available interpreter resources.

### Healthcare delivery for patients with COVID-19

We found several themes that described the critical changes required to newly provide care for surges in patients with COVID-19. These included organizing clinical spaces and supplies, newly caring for hospitalized adults, and testing patients and staff for COVID-19.

#### Organizing clinical spaces and supplies

Hospitals reorganized clinical spaces to accommodate surges in adult patients and minimize patient and staff COVID-19 exposure. They also had to ensure adequate personal protective equipment, testing supplies, and medications/devices. This was often coordinated across hospital networks, cities, or states. Spaces were created/modified in several ways, including converting shared rooms to single occupancy and creating COVID-19 isolation units or city-designated COVID-19 hospitals. Community hospital pediatric units sometimes closed and transferred all children to children’s hospitals in an effort to increase adult capacity. Participants voiced concern that this led to decreased care continuity, transportation burdens, and challenges in maintaining community hospital clinicians’ skills/competencies.

*"Being in a [hospital] network and working together … helped us maintain at least a basic level of what we needed.”* –Physician administrator, Hospital 7

*“We closed all pediatric floors and centralized all pediatric admissions to the [children’s] hospital … [It] was very disruptive to our working relationship with the nurses and maintaining pediatric competencies.”* –Frontline physician, Hospital 2

Key Lessons: Hospitals should consider leveraging relationships across hospital networks/cities/states to obtain supplies. In addition, hospitals should consider establishing a timeline for periodic re-assessment of space reorganization plans (through review of hospital operations and community prevalence data).

#### Newly caring for hospitalized adults

Adult-medicine trained outpatient clinicians, pediatric inpatient clinicians, and trainees were deployed to newly care for hospitalized adult patients during surges. Clinicians were supported in this new scope of work via integration into teams supported by adult-trained residents and attendings as well as provision of just-in-time trainings and educational resources (e.g., from the multi-institutional POPCoRNetwork) [18]. It was important for patient safety to define exclusion criteria for adult patients cared for by pediatric clinicians, in order to reduce overall acuity/complexity when possible.

*“Those who deployed pediatric providers to adult units described staffing adult teams to ensure at least one provider was adult-trained, including the creation of a “super-hospitalist” role to oversee multiple teams.”* –Physician administrator, Hospital 10

Key Lessons: When surging patient volumes demand staffing clinicians from other disciplines, hospitals can support clinicians, and support patient safety, through 1) the creation of integrated, mixed specialty care teams; 2) defining patient exclusion criteria to limit patient acuity/complexity; and 3) providing targeted educational resources and just-in-time trainings.

#### Testing patients and staff for COVID-19

Participants described many challenges to COVID-19 testing, including limited access to testing supplies, delays in obtaining test results, and rapidly changing evidence/policies. Delays in test results risked COVID-19 exposures for staff and patients and complicated care planning (e.g., delays in procedures/operations).

*“Some [parents] have been pretty frustrated with the fact that … [they] need to be COVID-tested, but they're not getting their results for, let's call it like four hours, potentially. But during that time, their kid has been moved into a room with another family, and they don't know the results of the test.”* –Caregiver, Hospital 5

Key Lesson: Hospitals should strive to optimize supplies and timely test results, possibly via working with other hospitals and/or community partners.

### Effects on hospital personnel

We found several themes that described impacts of the COVID-19 pandemic on hospital personnel, including trainees. These themes included navigating staffing challenges, impacting mental health, and disrupting trainee education.

#### Navigating staffing challenges

Participants described a variety of staffing challenges including: 1) being overstaffed due to declines in pediatric inpatient volumes (at times necessitating staff furlough); 2) facing staffing shortages due to illness and need for quarantine; and 3) deploying staff to newly care for hospitalized adults during surges. Participants described the need for flexible, frequently re-evaluated staffing models as well as extra backup coverage plans. Some participants expressed frustration that deployment to adult services was considered “volunteer” work without a clear plan for repayment.

*“For a lot of reasons, volumes were down … Because of that, we were overstaffed with providers at times, yet still feeling like we’re waiting for this surge … Some of our teams were collapsed. Roles were collapsed.”* –Front-line physician, Hospital 3

Key Lessons: Hospital leaders should consider frequently re-evaluating staffing models; creating/enhancing backup staffing plans; and being transparent about salary changes, furloughs, and reimbursement.

#### Impacting mental health

The pandemic negatively impacted the mental health of clinicians and trainees, and hospital leaders tried to create supports to address this. Participants described a confluence of many stressors, including fear of becoming infected with COVID-19 and infecting others, high stress/low morale among co-workers (including trainees), and longer work hours. Those newly caring for hospitalized adults additionally experienced higher illness acuity/dying patients and distress from feeling unprepared to safely care for adult patients. Participants were concerned about increases in anxiety, depression, and post-traumatic stress disorder.

*“I think everybody felt very stressed, very anxious. People were worried about bringing COVID home to their families, worried about getting their kids sick, worr [ied] about dying…” –*Front-line physician, Hospital 6

*“She had a patient die every day … The psychic injury from doing this … is really, really real. I worry about PTSD.”* –Hospital administrator, Hospital 10

Hospital leaders responded by providing counseling services and hotlines, support groups, in-hospital wellness spaces, and other supports (e.g., temporary housing, childcare, transportation, financial assistance). Participants also described that robust infection control policies and access to needed supplies (e.g., PPE, testing supplies) reduced fear among staff.

Key Lessons: Hospitals should consider offering mental health services and other supports (e.g., temporary housing) to clinicians, including trainees and limit consecutive hours/days worked to avoid trainee/staff burnout.

#### Disrupting trainee education

Participants described concerns about the dramatic reductions in trainee participation in clinical activities, including total exclusion of medical and nursing students, and reductions in residents/fellows in pediatric clinical settings. Additionally, decreases in pediatric patient volumes and physical distancing practices (e.g., videoconference rounds) greatly limited patient interaction. Didactic education was also modified from in-person to videoconference.

*“As hospitalists, we do family-centered rounds, and that’s a way that we teach our trainees, and with COVID and preserving PPE, students were pulled out … I think it really impacted the medical students to not be at the bedside, examining patients, being part of the team.”* –Front-line physician, Hospital 7

Key Lesson: When crises limit in-person learning, training programs can support ongoing education through thoughtfully designing video-based curricula. Special attentions should be paid towards evaluating educational outcomes and trainee competencies.

## Discussion

In this national qualitative study, we found that the COVID-19 pandemic resulted in major changes in the care of hospitalized children. Hospital leaders rapidly implemented new policies, and this was facilitated by rapid synthesis of evolving internal and external data, development of command centers, and multidisciplinary stakeholder engagement. Hospital leaders optimized communication through regular, transparent, multi-modal, and bi-directional communication. Videoconference and telehealth use greatly increased to promote physical distancing; however, participants described potential negative impacts on trainee education and communication with non-English speaking patients. Surging adult censuses and low pediatric volumes required clinicians to newly care for hospitalized adults. This was facilitated by developing care teams supported by adult hospitalists, multidisciplinary support via videoconference, and educational resources. In some cases, community hospital pediatric units closed to expand bed availability for adult patients. Finally, participants described negative impacts on clinician mental health and applauded expanded mental health resources and wellness activities/spaces.

Our findings align with prior studies. A survey study by the Department of Health and Human Services identified challenges in healthcare delivery during the COVID-19 pandemic, including PPE shortages, inadequate testing supplies and resulting delays, staffing challenges, difficulty maintaining bed capacity, and decreased revenue [19]. Additionally, prior editorials emphasized the importance of several areas of pandemic response, including centralized, multidisciplinary leadership, frequent bi-directional communication, backup staffing plans, wellness resources, and supports for clinicians newly caring for hospitalized adults [20, 21]. Our study is unique in providing more in-depth analyses of these and additional areas, as well as integrating the perspectives of pediatric leaders and clinicians from community hospitals and those of caregivers.

Although closure of community hospital pediatric units may have increased capacity to accommodate adults [6, 22], our participants described important potential drawbacks to this approach, including disruptions in patient care continuity, transportation burdens for families, decreased hospital revenue/furloughs, and difficulties maintaining clinicians’ pediatric competencies. These closures exacerbated pre-pandemic threats to the viability of pediatric units in community hospitals, including: 1) increasing consolidation of pediatric care in specialized/children’s hospitals; 2) financial instability; 3) fluctuating and overall low volumes; 4) limited access to pediatric services (e.g. radiology); 5) hospital leadership focus preferentially on adult services; and 6) disadvantageous payer policies [23–27]. Krugman et al. assert that key supports for ensuring the viability of pediatric units in community hospitals are employment of pediatric hospitalists by affiliated children’s hospitals and increased access to sub-specialty consultation via telemedicine [27]. Our findings indicate the importance of financially supporting these units in re-opening and frequent, data-driven, re-assessment of unit closures in future crises.

During the COVID-19 pandemic, surges in adult patient volumes led to clinicians newly caring for hospitalized adults. This was done via clinician deployment to adult units and/or care of adults in pediatric units. Participants described challenges with both approaches, but highlighted strategies for supporting clinicians practicing outside their scope of practice, including access to adult-trained clinicians and educational resources. Prior editorials assert that it is preferable to adapt pediatric units to care for adults because this approach provides clinicians that are practicing out of their scope with familiar settings, routines, and relationships (which enhance trust and teamwork) [28, 29]. Suggested key strategies include involvement of multidisciplinary adult stakeholders in planning (e.g. pharmacy), adoption of adult protocols/order sets, safety huddles, safety event reporting, a system for adult care escalation, and access to adult hospitalist and sub-specialty consultation [8, 28, 29].

We found participants were deeply concerned about negative impacts of the pandemic on clinicians’ mental health, including trainees. They described increases in anxiety, depression, and PTSD due to fear of COVID-19 infection, uncertainty, lack of normalcy in routines, high illness acuity/dying patients, and practicing outside one’s scope. Such impacts on clinician mental health may cause declines in quality of care and access for patients (as clinicians exit the workforce). Consequently, hospitals and training programs must be proactive in supporting clinician/trainee mental health [30]. Devaraj et al. emphasize that hospitals must ensure “psychological safety” by ensuring a non-threatening environment that demonstrates openness, trust, recognition, and gratitude [31]. Hospital and educational leaders should consider long-term continuation and expansion of mental health services and supports that were developed during the pandemic.

We found major disruptions in trainee education during the COVID-19 pandemic. There were reductions in clinical training opportunities due to limitations in in-person learning and declines in pediatric patient volumes. Educators tried to meet learner needs through expansion of virtual learning opportunities [32, 33]. Despite this, participants expressed worry that education quality declined. Ultimately, the downstream effects of these educational disruptions are unknown, raising concern about how we can ensure adequate clinical competencies of graduating trainees. Our study highlights the need to frequently assess trainee skills and adapt educational strategies/create catch-up opportunities as needed.

Given that our study was qualitative, we cannot assert causality between the pandemic and the described changes in pediatric hospital care. Further quantitative and longitudinal investigation is needed to confirm our findings (e.g., negative mental health impacts). Also, our findings may not apply to hospitals with lower or later peaks in COVID-19 incidence. The parents/caregivers interviewed in this study were all English-speaking members of family advisory councils from children’s hospitals; thus, their views may not be broadly generalizable. Lastly, participants from additional disciplines (e.g., pharmacy) and trainees may contribute important perspectives and represent an important focus of future study.

In conclusion, we identified several major changes in inpatient pediatric care delivery during the COVID-19 pandemic, including development of new policies to promote physical distancing and limit exposure, broad application of video technology, initiation of COVID-19 testing, and reorganization of care spaces and staffing models. Community hospital participants described unique challenges related to communication with leadership, maternal-newborn care, pediatric unit closures, and COVID-19 testing and supplies. We also outline potential lessons to guide hospitals leaders in future crises, including strategies for successfully developing new hospital policies, effectively communicating with staff, and supporting clinicians’ expanding scope of practice. Lastly, we highlight potentially important areas of focus for pandemic recovery, including assessing and supporting clinicians’ mental health and well-being, re-evaluating trainees’ skills/competencies, and adapting educational strategies as needed.

## Supplementary Information

**Additional file 1: Table S1.** Clinician/Administrator Interview Guide. **Table S2.** Parent/Caregiver Interview Guide.

## Data Availability

The datasets used and/or analyzed during the current study are available from the corresponding author on reasonable request.

## Abbreviations

COVID-19: Coronavirus disease 2019
PPE: Personal protective equipment
