# Supplementary material for: Changes in pediatric hospital care during the COVID-19 pandemic: a national qualitative study
Source: BMC Health Serv Res. 2021 Sep 11;21:953. doi: 10.1186/s12913-021-06947-7 (PMC8435183; doi:10.1186/s12913-021-06947-7)
Supplement: Supplementary file 1 — Additional file 1: Table S1. Clinician/Administrator Interview Guide. Table S2. Parent/Caregiver Interview Guide. [file 12913_2021_6947_MOESM1_ESM.zip › Penwill_Appendix Table 1_BMC.docx]

**Table S1: Clinician/Administrator Interview Guide**

| ***Open-Ended Questions*** | ***Potential Probes***  *(selected based on responses to open-ended questions &*  *evolving conceptual model)* |
| --- | --- |
| Can you tell me about your role and responsibilities at the hospital? |  |
| Can you describe some of the problems your hospital faced during the COVID-19 pandemic and the changes that took place to address those problems? We are specifically interested in changes that affected care in inpatient pediatric or newborn units. | - Can you describe who was involved in your hospital’s efforts to plan for and respond to the COVID-19 pandemic? - How did leaders at your hospital monitor local and national COVID-19 surveillance data? - Can you describe how new hospital policies for things such as COVID-19 screening, testing, and management were developed? How was the most current evidence reviewed and incorporated? - Can you describe how hospital staff received communication about changes in hospital care and policies during the COVID-19 pandemic? How was the highest priority information emphasized? - What changes were made in patient care to prepare for and manage surges in patients ill with COVID-19? - How rapidly were hospital staff able to implement the changes planned by hospital leaders? - Can you describe any disaster plans your hospital already had in place? - If your hospital had disaster plan policies, how were these revised during the COVID-19 pandemic? - Did your hospital staff have any types of emergency drills regarding viral outbreaks prior to COVID-19? - What resources did you need to develop to support your activities during this time? - How were supplies of needed equipment, such as personal protective equipment and COVID-19 testing supplies, procured and maintained during the COVID-19 pandemic? - Can you describe any changes that were made to address financial concerns during the pandemic? - Can you describe any ways in which staffing plans were changed during the COVID-19 pandemic to ensure adequate staff were available for surges in patient volume? - Can you describe an example of how hospital staff were educated or trained in new skills during the COVID-19 pandemic, such as pediatric providers extending to provide adult care? - Can you describe an example of how trainee education was changed or affected? - How was the mental health of clinicians affected? - What resources were made available to help support staff during the COVID-19 pandemic, such as housing or mental health supports? Can you describe if and how these were helpful? |
| In what ways did your hospital’s response to COVID-19 go well? | - What resources were valuable in planning these changes? How were they helpful? - What policies or resources did you already have in place that made things easier? - How did hospital leaders and staff work together during the pandemic? |
| What bumps in road did you encounter in your hospital’s response to COVID-19? What was learned from these experiences? | - Can you describe an example of a change that did not go well? How did your hospital or unit respond, and what was learned from this experience? |
| Can you describe any examples of how changes in hospital care during the COVID-19 pandemic affected vulnerable groups of children, such as children from poor or racial minority populations, or non-primary English speakers? | - Can you describe changes in clinical care workflows that may have adversely affected vulnerable groups? |
