# Supplementary material for: Changes in pediatric hospital care during the COVID-19 pandemic: a national qualitative study
Source: BMC Health Serv Res. 2021 Sep 11;21:953. doi: 10.1186/s12913-021-06947-7 (PMC8435183; doi:10.1186/s12913-021-06947-7)
Supplement: Supplementary file 1 — Additional file 1: Table S1. Clinician/Administrator Interview Guide. Table S2. Parent/Caregiver Interview Guide. [file 12913_2021_6947_MOESM1_ESM.zip › Penwill_Appendix Table 2_BMC.docx]

**Table S2: Parent/Caregiver Interview Guide**

| ***Open-Ended Questions*** | ***Potential Probes*** |
| --- | --- |
| Can you describe your interactions with the children's hospital during the COVID-19 pandemic, as a parent/caregiver of a child admitted to the hospital and as a family advisory board member? |  |
| Can you describe any changes you noticed in hospital care practices that might have been related to the pandemic? | - Access to care - Communication with your care team - Rounding practices - Family visitation policies - Infection Control & Family PPE policies - Inpatient resources & supports (e.g., Child Life, Social Work) |
| Can you describe an example of a way in which your hospital experience went well? |  |
| Can you describe an example of a way in which your hospital experience could have been better? |  |
| Were you ever concerned that these changes may have impacted your child's safety or quality of care? How so? |  |
| Through the family advisory council, what feedback have you heard from other parents? |  |
